# Current Hospital Topics

**Published:** 1907-01-05

**Authors:** 


### HOSPITAL ADMINISTRATION.

### CONSTRUCTION AND ECONOMICS.

### CURRENT HOSPITAL TOPICS.

#### Bolingbroke Hospital.

The Rev. Canon Erskine Clarke, the chairman and also the founder of this hospital, has issued an urgent appeal for £14,000 to complete the Building Fund for the new wards, the foundation-stone of which was laid in May last by H.R.H. the Princess Royal. Canon Clarke states that the present accommodation is totally inadequate, and serious cases frequently have to be refused admission because there is no room to receive them. When completed the new hospital will contain 153 beds, and the necessity for the present enlargement has been very forcibly impressed upon the Governors by the Committee of King Edward's Hospital Fund, who have subscribed generously towards it. The sum urgently needed is £14,000, and there ought to be no difficulty in raising this without delay. The hospital is situated in one of the most densely populated and poorest districts of South-west London, where a modern and up-to-date general hospital is undoubtedly required. Contributions should be sent to the Secretary of the Building Committee at the hospital, Wandsworth Common, S.W.

#### Colonial Contrasts.

An incident occurred at a recent meeting of the Hobart General Hospital which reads strangely to a resident in the old country. A bequest of £1,600 had been left to the hospital by Amy Ball, but the will was disputed by the relatives, and a compromise was effected through the Solicitor-General, as a result of which the hospital received £800 only. After an explanation by the Chairman, in the course of which he stated that the Premier had promised to bring the matter before the Executive, the Mayor expressed doubts as to the legality of the proceedings, and urged that the hospital should obtain a written recommendation from the Solicitor-General to accept the course mentioned and the Premier's written confirmation of this procedure. That would, he thought, place the matter on a business footing. The Mayor assured the meeting that if the Premier confirmed the advice of the Solicitor-General he would be quite ready to agree to what had been done. Thereupon the chairman, whilst complimenting the Mayor upon the efficient discharge of a public duty, explained that the compromise had been arrived at after months of negotiations by a special committee in full consultation with the Solicitor-General, and that the judge had signed the order embodying the compromise, which could not be upset. The life of a Premier is not invariably a happy one, but if he is to have the care of all the details of the financial work of the hospitals thrust on his shoulders, we imagine there are few men who would consent to hold the position long, even in a British Colony.

#### Hospital Saturday Fund.

The *Hospital Saturday Fund Journal* is largely taken up with matters relating to the resignation of Mr. W. G. Bunn, the organisation of the office, and the appointment of new secretaries. We are glad to mark that the warmest testimony was paid by Sir Savile Crossley, chairman, Mr. R. B. D. Acland, K.C., who has done so much for the Hospital Saturday Fund, Dr. T. D. Lister, and by many members of the Council, and the members of the official staff of the Fund. For seventeen years Mr. Bunn rendered devoted service to the Hospital Saturday movement, and he has well deserved this praise and recognition. The Board of delegates have decided to present to Mr. Bunn a testimonial, to consist of a purse of money and an illuminated address. Contributions may be sent to Mr. A. W. Davies, 54 Gray's Inn Road, W.C. The testimonial will be presented at the annual dinner of the Fund to be held shortly. The official staff, in expressing their good wishes for his welfare in the future, presented Mr. Bunn, on leaving, with a clock as a token of their esteem and affection. Mr. William Jolly, after seventeen years' service, has resigned his post as assistant organising secretary, owing to advanced age, and has received a gratuity from the Board on his retirement. Mr. W. H. Reed is now the assistant organising secretary: Mr. E. Radford has been appointed organising secretary, having been selected from 75 applicants for the post. We have further to record the resignation of Mr. W. V. Stratford, chairman of the Collection Committee of the Fund, after 25 years' zealous service in various positions, which Mr. Stratford describes as the happiest part of his life. Mr. Stratford's resignation was accepted with the deepest sorrow and regret by his colleagues, for his work had called forth the admiration of every member of the Southwark Committee of which he was honorary local secretary. The Hospital Saturday Fund, apart altogether from the money it has raised for the hospitals, has done a very real service by arousing the interest of a large and increasing number of men and women, who have devoted their leisure to the work of helping the hospitals. We are glad to learn that it is hoped the amount collected by this Fund for the current year will be the largest on record.

